# Ellagitannins of the fruit rind of pomegranate *(Punica granatum) *antagonize *in vitro *the host inflammatory response mechanisms involved in the onset of malaria

**DOI:** 10.1186/1475-2875-9-208

**Published:** 2010-07-19

**Authors:** Mario Dell'Agli, Germana V Galli, Michela Bulgari, Nicoletta Basilico, Sergio Romeo, Deepak Bhattacharya, Donatella Taramelli, Enrica Bosisio

**Affiliations:** 1 Dipartimento di Scienze Farmacologiche, Università degli Studi di Milano, Via Balzaretti, 9 - 20133 Milano, Italy; 2 Dipartimento di Sanità Pubblica-Microbiologia-Virologia, Università degli Studi di Milano, Italy; 3 Dipartimento di Scienze Farmaceutiche "Pietro Pratesi", Università degli Studi di Milano, Italy; 4 Oddisi Research Laboratory, Kedar Gouri Road Bhubaneswar, 751002-India

## Abstract

**Background:**

The sun-dried rind of the immature fruit of pomegranate (*Punica granatum*) is presently used as a herbal formulation (OMARIA, Orissa Malaria Research Indigenous Attempt) in Orissa, India, for the therapy and prophylaxis of malaria. The pathogenesis of cerebral malaria, a complication of the infection by *Plasmodium falciparum*, is an inflammatory cytokine-driven disease associated to an up-regulation and activity of metalloproteinase-9 and to the increase of TNF production. The *in vitro *anti-plasmodial activity of *Punica granatum (Pg) *was recently described. The aim of the present study was to explore whether the anti-malarial effect of OMARIA could also be sustained via other mechanisms among those associated to the host immune response.

**Methods:**

From the methanolic extract of the fruit rind, a fraction enriched in tannins (*Pg*-FET) was prepared. MMP-9 secretion and expression were evaluated in THP-1 cells stimulated with haemozoin or TNF. The assays were conducted in the presence of the *Pg*-FET and its chemical constituents ellagic acid and punicalagin. The effect of urolithins, the ellagitannin metabolites formed by human intestinal microflora, was also investigated.

**Results:**

*Pg*-FET and its constituents inhibited the secretion of MMP-9 induced by haemozoin or TNF. The effect occurred at transcriptional level since MMP-9 mRNA levels were lower in the presence of the tested compounds. Urolithins as well inhibited MMP-9 secretion and expression. *Pg*-FET and pure compounds also inhibited MMP-9 promoter activity and NF-kB-driven transcription.

**Conclusions:**

The beneficial effect of the fruit rind of *Punica granatum *for the treatment of malarial disease may be attributed to the anti-parasitic activity and the inhibition of the pro-inflammatory mechanisms involved in the onset of cerebral malaria.

## Background

Pomegranate *(Punica granatum *L., Punicaceae) is used in the traditional medicine of different Asian cultures for the treatment of a variety of ailments. In Ayurvedic medicine, the plant, described under its Sanskrit name "dalima" (fruit), is considered as a "blood tonic" and used to cure parasitic infections [1]. The decoction of the root was found beneficial in fevers and chronic debility due to malaria. Moreover, the fruit rind powder was found to possess immunomodulatory properties [2].

The eastern province of Orissa (India) is an area endemic for both *Plasmodium falciparum *and *Plasmodium vivax*; malaria constitutes a major health problem for the population, in particular for those living in rural areas. Since 1998, malaria patients referring to the Ayurveda dispensary receive a herbal preparation named OMARIA, made of sun-dried rind of the immature *P. granatum *fruits (*Pg*). OMARIA (the acronym for Orissa Malaria Research Indigenous Attempt) is distributed as a home based economic remedy for prophylaxis under the banner "Fight Malaria At Home/*Ghare Maro Malaria*. Clinical application started in 1998 by the Indian Red Cross Society Charitable Ayurveda dispensary (c/o District Magistrate Koraput) on behalf of D. Bhattacharya. Dispensary records indicate that OMARIA can successfully control *P. falciparum *and *P. vivax *infections in all patients including infants and pregnant women [3,4]. OMARIA is administered as gelatine capsules (courtesy of m/s Sunil Health Care Ltd. New Delhi, India), containing each 825-850 mg of *Pg*. The therapeutic dose is one capsule every eight hours for three consecutive days. For prophylaxis, one capsule has to be taken in every day (children receive half the dosages) for a period ranging between two or four weeks/six months. Records from the Ayurveda-Indian Red Cross Society indicate a positive impact on the health status of the population under OMARIA coverage: the prophylactic intervention appears not only to reduce malaria episodes, but also the incidence of other infectious diseases, such as measles, chicken pox and conjunctivitis [4].

The reported anti-malarial effectiveness of the OMARIA was attributed to the anti-parasitic activity of a fraction enriched in tannins (*Pg-*FET) obtained from the *Pg *methanolic extract [5]. The effect could be attributed to different constituents of *Pg*-FET, namely ellagic acid (EA), and punicalagin, which inhibited *in vitro *the growth of *Pf *asexual blood stages [5,6]. Whether *Pg *preparations could help to control the malarial disease by adjuvant mechanisms, as well, remains unexplored. The present research was undertaken with the aim of testing the effects of Pg preparations on the pathways involved in the onset severe malaria, which may develops during *Pf *infection.

It is largely accepted that severe malaria is an inflammatory cytokine-driven disease. There is remarkable evidence that tumour necrosis factor (TNF) and interleukin-1 are important contributors to the systemic disease caused by the infectious agent *Pf *[7,8].

The innate immune system seeks to destroy the protozoa and remove the remnants via phagocytosis by monocytes and neutrophils. Circulating levels of TNF, a mediator of the innate immune system, are increased after infection, as a consequence of stimulation of monocyte-macrophages by infected red blood cells (IRBC) or phagocytosis of haemozoin (Hz, the malarial pigment) phagocytosis by human monocytes [9-12]. TNF in turn enhances the synthesis of metalloproteinase-9 (MMP-9) in monocytes and macrophages [9,10]. More recently, it has been shown that human monocytes fed with Hz or IRBC display increased MMP-9 activity and protein/mRNA expression and increased production of TNF and a role of MMP-9 and TNF in the onset of cerebral malaria has been postulated [11,12].

The wide-range of therapeutic benefits of pomegranate have been attributed to its anti-oxidant and anti-inflammatory properties. It was shown that *Pg *fruit juice and its constituents ellagitannins, have a significant and broad inhibitory effect on MMPs, including MMP-9 [13,14,1]. In addition, *Pg *fruit juice and ellagitannins suppress inflammatory cell signalling induced by TNF in colon cancer cells [15].

However, it was not clear which of the compounds present in *Pg*-FET or ellagitannins could antagonize the host inflammatory response. In the present study, it has been investigated whether *Pg*-FET, EA, and punicalagin, the compounds previously identified in *Pg*-FET, inhibited MMP-9 upregulation and secretion in THP-1 cells induced by native haemozoin (Hz) and TNF. Since ellagitannins were shown to be converted by the human gut microflora into urolithin A, B and urolithin-8-methylether [16-20] these metabolites were also evaluated for their inhibitory effect.

Here, it is shown that *Pg*-FET and its constituents EA and punicalagin, all inhibited the MMP-9 secretion and expression in THP-1 cells, fed with Hz or TNF. *Pg*-FET and individual compounds were also able to inhibit MMP-9 promoter activity after stimulation with Hz. The inhibitory effect was partially due to the inhibition of NF-κB pathway. Urolithins were also active. It is then plausible that *Pg*, in addition to the direct effect on the parasite, modulates the malarial disease via the inhibition of the inflammatory response induced by haemozoin.

## Methods

### Chemicals

Amberlite XAD16 resin was purchased from Sigma-Aldrich (Milan-Italy) and Kollidon^® ^from BASF Chemical Company (Germany). Ellagic acid (EA) was acquired from Fluka-Sigma-Aldrich (Milan, Italy), punicalagin was obtained from AvaChem Scientific LLC, San Antonio, Texas, USA. Urolithin A, B and urolithin A-8-methylether were synthesized as described [5]. All reagents were purchased from Fluka-Sigma-Aldrich (Milan, Italy).

### Plant material and extract fractionation

The pomegranate immature fruit was manually plucked from plants growing in tropical forest area of India. The fruits were cut and the arils discarded. The rind was sun-dried, finely ground, delipidized by petroleum ether (40:60) and extracted twice with methanol (MeOH, 1 g/10 ml). The w/w yield of the MeOH extract was 38% with respect to the starting crude material. For the tannin removal, the extract was treated with Kollidon^® ^(1:25, w/w) thus obtaining *Pg*-MeOH-DT.

For the preparation of a fraction enriched in tannins (*Pg*-FET), 5 g of the *Pg*-MeOH extract were dissolved in 500 ml of water and chromatographed on Amberlite XAD16 resin as described [5]. The w/w yield of *Pg*-FET was 60% with respect to the *Pg*-MeOH extract. *Pg*-FET was analyzed by HPLC-MS as described [5].

### Purification of native haemozoin

*Plasmodium falciparum *cultures were carried out according to the method of Trager and Jensen with slight modifications [21]. Briefly, a chloroquine (CQ)-sensitive *P. falciparum *strain (D10) was maintained at 5% haematocrit at 37°C in complete culture medium (RPMI 1640 supplemented with NaHCO_3 _24 mM, 1% AlbuMax II, 0.01% hypoxanthine, 20 mM HEPES, and 2 mM glutamine). All cultures were maintained in a standard gas mixture, consisting of 1% O_2_, 5% CO_2_, 94% N_2_.

To separate haemozoin, cells were washed twice with serum-free culture medium, resuspended to 25% haematocrit, and fractionated onto a discontinuous Percoll/4% sorbitol (w/v) gradient (0, 40, 80%). After centrifugation at 1075 *g*, native haemozoin was collected from the top of the gradient, 0-40% interphase. Native haemozoin was washed three times with PBS and stored at 20% (v/v) in PBS at -20°C. The haem content of HZ was determined by building a standard curve with increasing concentrations of haemin (0-100 μM) and measuring absorbance at 405 nm of a weighed amount of the compound dissolved in 0.1 M NaOH.

### Cell cultures

Human THP-1 monocytic leukaemia cells (ATCC, Teddington, UK) were grown in RPMI 1640 (Invitrogen s.r.l., Milano, Italy) supplemented with 100 units penicillin/ml, 100 μg streptomycin/ml, 10 mM HEPES, 1 mM Sodium Pyruvate, 0.05 mM beta-mercaptoethanol, and 10% heat-inactivated foetal calf serum (FCS, Euroclone, Milano, Italy).

*Pg*-FET was used at 50-100 μg/ml. Concentrations were chosen by taking into consideration the daily dosage of P.g. (2550 mg/die), the recovery of the MeOH extract, the percentage of FET in the MeOH extract and assuming complete absorption.

*Pg*-FET and pure compounds were used at no toxic concentrations. Cytotoxicity was evaluated by testing alterations of mitochondrial functionality as assessed by MTT test [22]. No sign of cytotoxicity was observed at concentrations ranging from 50 to 250 μg/ml for FET and 25 μM for individual compounds.

To evaluate the effect of *Pg*-MeOH extract on the MMP-9 secretion in PMA-treated THP-1, 1.2 × 10^6 ^cells/ml were plated in 24-well plates in the presence of 10 nM PMA to induce differentiation to macrophages. After 48 hours, cells were treated with the extract for 24 hours by using RPMI 1640 deprived of FCS, and the medium collected and subject to zymography.

To evaluate the effect of the fraction/compounds on TNF-induced MMP-9 secretion, THP-1 cells were plated in 96-well plated (1 × 10^6 ^cells/ml) in the presence of 10 ng/ml TNF, and treated with the compounds under study or the only vehicle (final concentration 0.1%) by using medium deprived of FCS. 48 hours after, cells were centrifuged and the media collected for zymography.

To evaluate the effect on HZ-induced MMP-9 secretion, THP-1 cells were plated in 96-well plates (1 × 10^6 ^cells/ml) in the presence of 6 μg/ml HZ and incubated at 37°C for four hours. Cells were centrifuged and resuspended in RPMI 1640 without FCS in the presence of the compounds under study or the vehicle. 48 hours after, cells were centrifuged and used for RNA extraction and protein measurement, whereas the media were subject to zymography.

### SDS-PAGE zymography

The gelatinolytic activity of MMP-9 secreted by THP-1 cells was evaluated as previously described [23]. Briefly, cells were exposed to TNF or HZ in the presence of the tested compounds. Epigallocatechin-3-gallate (EGCg) 20 μM was used as reference inhibitor of MMP-9 expression [23]. Control samples received the vehicle only. Aliquots of conditioned medium underwent electrophoresis on 7.5% polyacrylamide gels containing 10% SDS and gelatin (1 mg/ml). The gels were then washed in 2.5% Triton X-100 (Sigma-Aldrich, Milan, Italy) at room temperature and then incubated overnight at 37°C (50 mM Tris pH 7.5 containing 150 mM NaCl, 10 mM CaCl_2_, 1 μM ZnCl_2_; activation buffer). At the end of the incubation, the gels were stained with Coomassie brilliant blue R-250 (Sigma-Aldirch, Milan, Italy). For the quantification of zymograms, densitometric scanning was performed using QuantityOne software (Bio-Rad) and each lysis area was normalized against intracellular protein content determined by Bradford assay [24].

### Real time RT-PCR

THP-1 cells were treated as described above. Total RNA was extracted with the RNeasy Mini Kit (Qiagen, S.p.A., Milan, Italy) according to the manufacturer's instructions. Total RNA was quantified using the Ribo Green RNA Quantitation Assay from Molecular Probes (Invitrogen, Milan, Italy). Aliquots corresponding to 1000 ng of total RNA were reverse transcribed by using the iScript™cDNA synthesis kit (Bio-Rad Laboratories, Milan, Italy) following the manufacturer's protocol. Aliquots of the cDNA were subject to real-time PCR with a SYBR Green kit (Bio-Rad Laboratories, Milan, Italy) following the manufacturer's instructions. 18 S rRNA was used as the housekeeping gene for sample normalization and was amplified in separate wells within the same plate. Primers for real-time PCRs were designed with Primer Express software (Applied Biosystems, Monza, Italy) and optimized to work in a two-step protocol. The oligonucleotides were synthetized by Primm (Milan, Italy) and the sequences are the following: MMP-9, forward primer 5'AAACCGAGTTGGAACCACGA3', reverse primer 5'TCAGGGAGACGCCCATTTC3'; 18 S rRNA forward primer 5'CGGCTACCACATCCAAGGAA3', reverse primer 5'AGGTAGTGACGAAAAATAACAATCACGG3'. The specificity of the amplified products was monitored performing melting curves at the end of each amplification reaction. All amplicons generated a single peak, thus reflecting the specificity of the primers. Dexamethasone was used as a reference inhibitor of MMP-9 expression (~80% inhibition at 100 nM).

### Plasmid construction

The DNA fragment corresponding to 1024 bp of the 5'-flanking region of the human MMP-9 promoter region (-1005 to + 19) cloned into the PGL3-basic vector and the corresponding mutant construction in the κB site (GGAATTCCCC to GtcAacagCC) [25] were a kind gift of Dr. Siro Simizu (Antibiotics Laboratory and Chemical Biology Department, Saitama University, Japan). pNF-κB-luc containing the luciferase gene under the control of three κB responsive elements of the promoter of the E-selectin gene was a kind gift of N. Marx (Department of Internal Medicine II-Cardiology, Ulm, Germany).

### Assay of NF-κB and MMP-9 promoter activity

The NF-κB and MMP-9 promoter activities were evaluated in 96-wells plates by means of transient transfection assays in THP-1 cells by using the DEAE-dextran method as previously described [26]. Briefly, cells were exposed to a mixture of DNA-dextran (750 μg/ml final concentration) for 30 min at 37°C, using 700 ng/ml NF-κB-luc and 500 ng/ml MMP-9-luc or mutated mκB-luc reporter plasmid/1.5 × 10^5 ^cells. Cells were then incubated in FCS-supplemented RPMI 1640 (final volume, 100 μl/well). 48 hours after cells were stimulated with HZ (6 μg/ml) for four hours and treated with the fraction/compounds under study, as described for MMP-9 secretion. 48 hours after, 100 μl/well Britelite plus (Perkin Elmer Waltham, MA, USA) was added and the plate read at the luminometer Victor™X3 Perkin Elmer 2030 (Perkin Elmer Waltham, MA, USA). Parthenolide (10 μM) was used as reference inhibitor of the NF-κB (~50% inhibition) and MMP-9 (~80% inhibition) promoter activity. Data are expressed as mean ± S.D. of triplicate samples.

### Statistical analysis

All experiments were reproduced at least three times and, where indicated, representative experiments are shown. Statistical analyses were performed with GraphPad Prism 5 software, using 1-way ANOVA test followed by Bonferroni's post hoc test. The significance was indicated as follows: * *p *< 0.05; ** *p *< 0.01; *** *p *< 0.001.

## Results

### Effect of Pg-FET on MMP-9 secretion and gene expression

**Figure 1:**
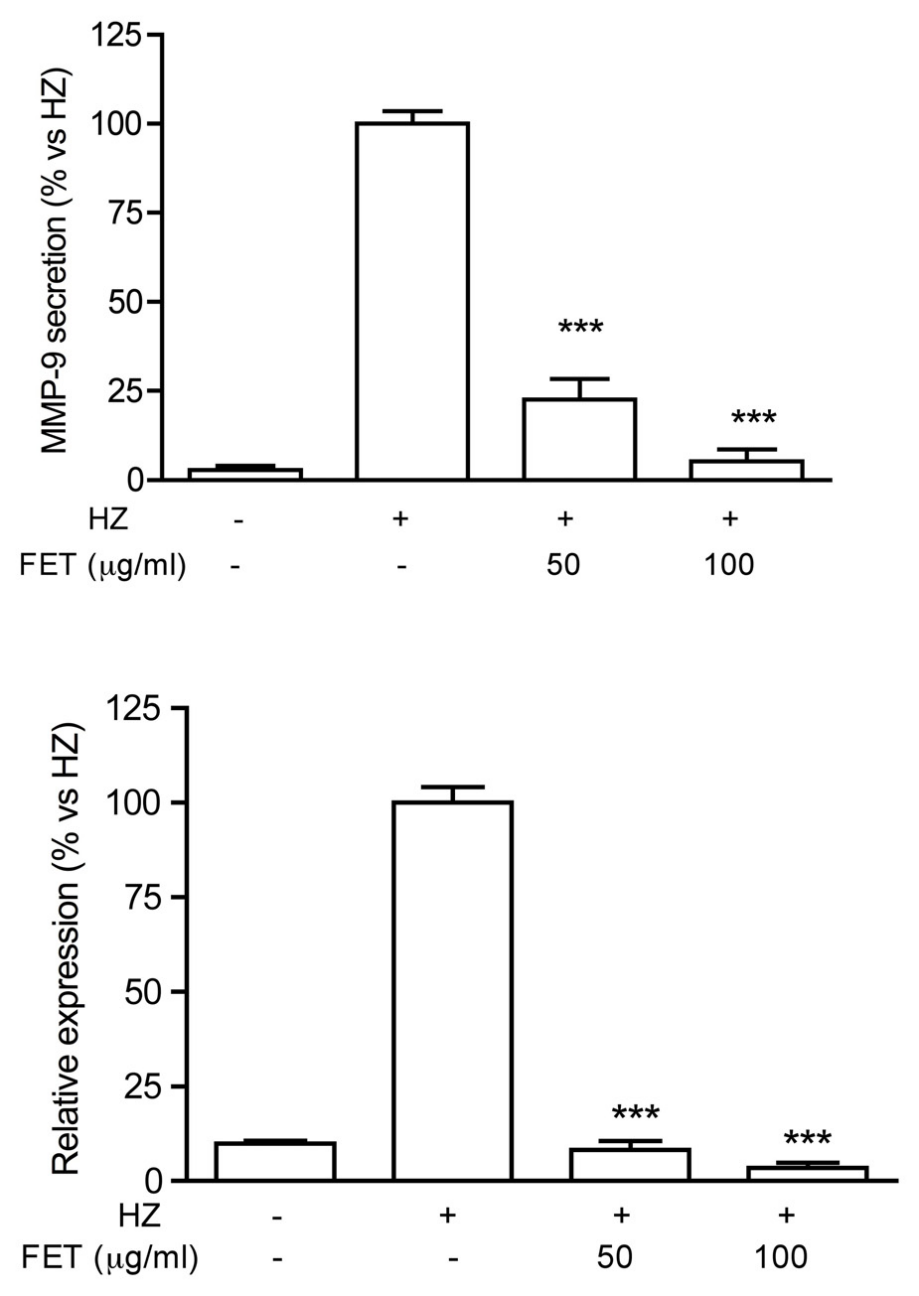
**Effect of *Pg*-FET on MMP-9 secretion (panel A) and mRNA levels (panel B) in THP-1 cells stimulated with Hz**. Pg-FET (50-100 μg/ml) antagonized the increase of Hz-induced MMP-9 secretion by 78% and 95%, respectively (**Panel A**), and the mRNA levels of 92% and 97%, respectively (**Panel B**). All experiments were reproduced at least three times. Statistical analyses were performed with GraphPad Prism 5 software, using 1-way ANOVA test followed by Bonferroni's post hoc test. The significance was indicated as *** *p *< 0.001.

**Figure 2:**
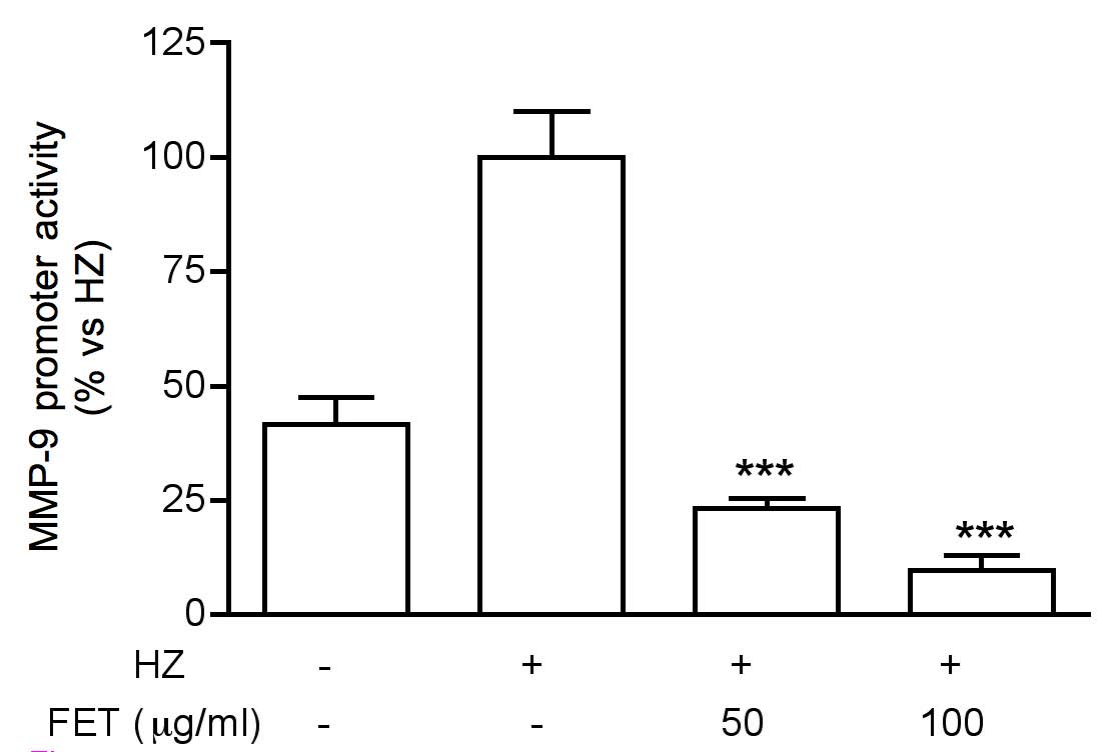
**Effect of *Pg*-FET on Hz-induced MMP-9 promoter activity**. Hz also induced a 2.5 fold increase of MMP-9 promoter activity, which was completely antagonized (80 and 90% inhibition) by *Pg*-FET at 50 and 100 μg/ml, respectively. Parthenolide (10 μM) was used as reference inhibitor of MMP-9 (~80% inhibition) promoter activity. Data are expressed as mean ± S.D. of triplicate samples. All experiments were reproduced at least three times. Statistical analyses were performed with GraphPad Prism 5 software, using 1-way ANOVA test followed by Bonferroni's post hoc test. The significance was indicated as *** *p *< 0.001.

In a preliminary experiment conducted in PMA-differentiated THP-1 cells, *Pg*-MeOH extract at 50 μg/ml inhibited the secretion of MMP-9 by 61%. When the extract was deprived of tannins, the inhibitory effect was dramatically reduced (-20%, not statistically significant vs controls). This result indicated that tannins were likely to be the active principles. Therefore a fraction enriched in tannins (*Pg*-FET) was prepared as described in the methodology section. To test *Pg*-FET and individual compounds, undifferentiated THP-1 cells were stimulated with Hz or TNF. The concentration of HZ used in the experiments (6 μg/ml) reflects the levels of Hz likely to be present in the plasma of infected patients [27]. Hz induced a significant increase of the MMP-9 mRNA levels and the amount of protein secreted by THP-1 cells compared to controls (Figure 1). *Pg*-FET, at 50 and 100 μg/ml, antagonized the increase of Hz-induced MMP-9 secretion by 78% and 95%, respectively, and the mRNA levels of 92% and 97%, respectively (Figure 1). Similarly, in cells stimulated by TNF, *Pg*-FET antagonized the increase of MMP-9 secretion by TNF, but the effect was significant only at the concentration of 100 μg/ml (-62%). As shown in Figure 2, Hz also induced a 2.5 fold increase of MMP-9 promoter activity, which was completely antagonized (80 and 90% inhibition) by *Pg*-FET at 50 and 100 μg/ml, respectively, thus confirming that the down-regulation of MMP-9 secretion was consequent to a decreased rate of MMP-9 gene transcription.

### Effect of punicalagin, EA and urolithins on MMP-9 secretion and gene expression

**Figure 3:**
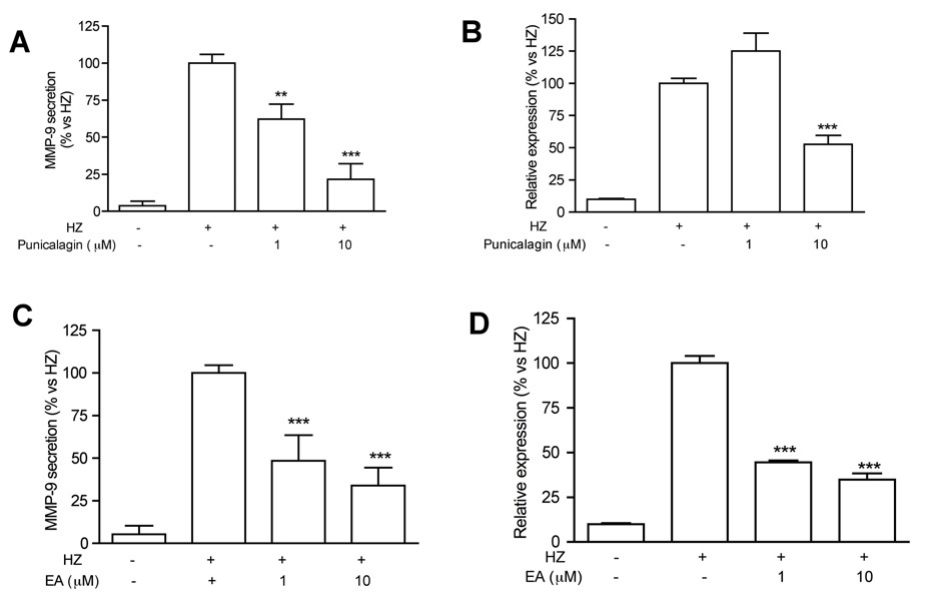
**Effect of punicalagin and EA on MMP-9 secretion (panel A, C) and mRNA levels (B, D) in THP-1 cells stimulated with Hz**. Punicalagin at 1 and 10 μM inhibited the release of MMP-9 in Hz stimulated THP-1 cells by 38% and 79%; the MMP-9 mRNA levels decreased accordingly (-47% at 10 μM; Figure 3A-B). EA at 1 and 10 μM inhibited the release of MMP-9 in Hz-stimulated THP-1 cells by 52 and 66%, respectively, and the MMP-9 expression by 56 and 65%, respectively (Figure 3C-D). All experiments were reproduced at least three times. Statistical analyses were performed with GraphPad Prism 5 software, using 1-way ANOVA test followed by Bonferroni's post hoc test. The significance was indicated as follows: ** *p *< 0.01; *** *p *< 0.001.

**Figure 4:**
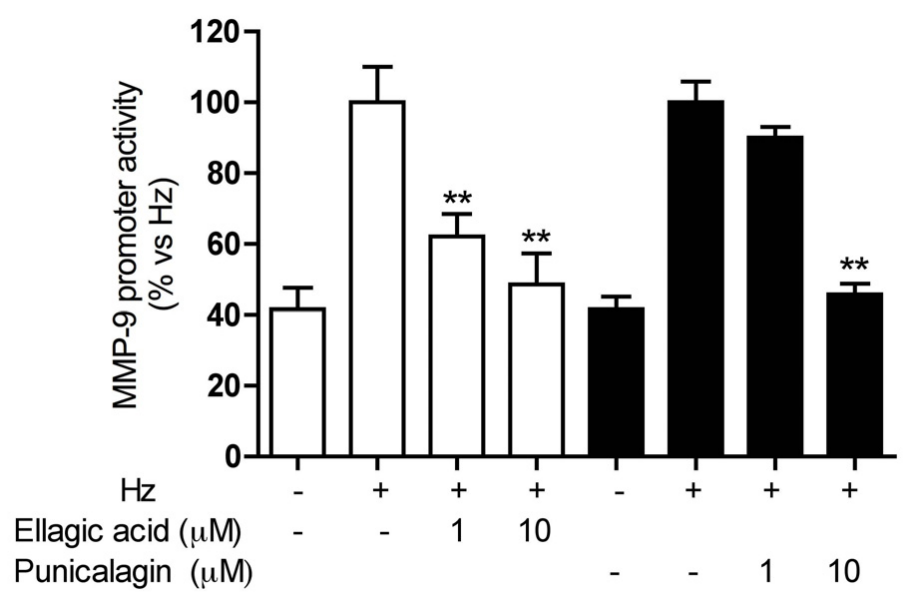
**Individual compounds EA and punicalagin inhibit Hz-induced MMP-9 promoter activity**. Ellagitannins were able to affect MMP-9 transcription since EA inhibited Hz-induced promoter activity by 38% and 50% at 1 and 10 μM, respectively, while the inhibitory effect of punicalagin was only statistically significant at 10 μM (-65%). Parthenolide (10 μM) was used as reference inhibitor of the MMP-9 (~80% inhibition) promoter activity. Data are expressed as mean ± S.D. of triplicate samples. All experiments were reproduced at least three times and, where indicated, representative experiments are shown. Statistical analyses were performed with GraphPad Prism 5 software, using 1-way ANOVA test followed by Bonferroni's post hoc test. The significance was indicated as ** *p *< 0.01.

As previously reported, EA and punicalagin represent the 13.4% and 29.1% of *Pg*-FET, respectively [5]. Punicalagin at 1 and 10 μM inhibited the release of MMP-9 in Hz stimulated THP-1 cells by 38% and 79%; the MMP-9 mRNA levels decreased accordingly (-47% at 10 μM; Figure 3A-B). EA at 1 and 10 μM inhibited the release of MMP-9 in Hz-stimulated THP-1 cells by 52 and 66%, respectively, and the MMP-9 expression by 56 and 65%, respectively (Figure 3C-D). Ellagitannins were also able to affect MMP-9 transcription since EA inhibited Hz-induced promoter activity by 38% and 50% at 1 and 10 μM, respectively, while the inhibitory effect of punicalagin was only statistically significant at 10 μM (-65%) (Figure 4).

**Figure 5:**
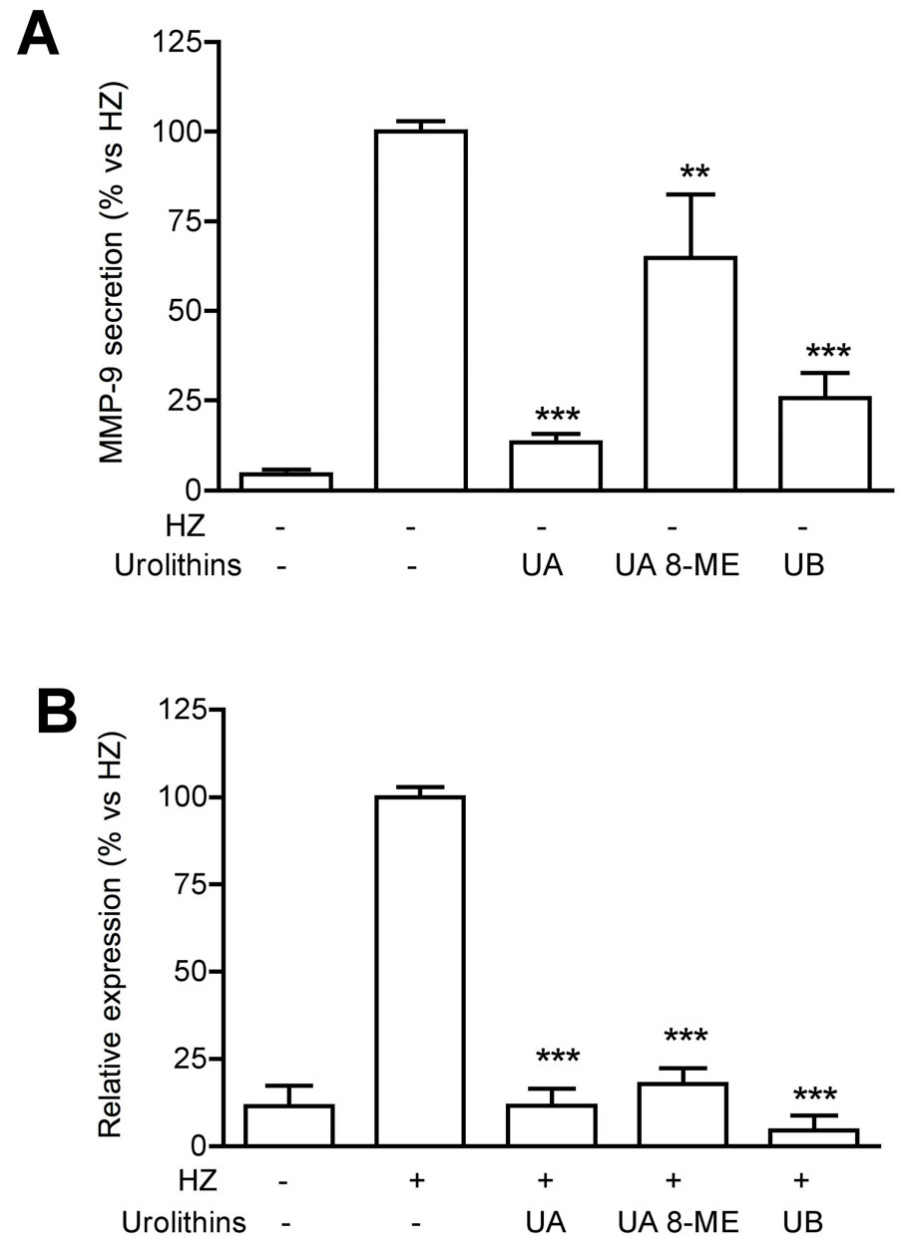
**Effect of urolithins on MMP-9 secretion (panel A) and mRNA levels (panel B) in THP-1 cells stimulated with Hz**. Urolithins A, B and 8ME at 25 μM inhibited the release of MMP-9 in Hz-stimulated THP-1 cells, urolithins A and B being the most active (-87, -37, and -74%, respectively; **Panel A**); the effect was associated to a decrease of MMP-9 mRNA levels (-88%, -95% and -82% respectively; **Panel B**). All experiments were reproduced at least three times. Statistical analyses were performed with GraphPad Prism 5 software, using 1-way ANOVA test followed by Bonferroni's post hoc test. The significance was indicated as follows: ** *p *< 0.01; *** *p *< 0.001.

**Figure 6:**
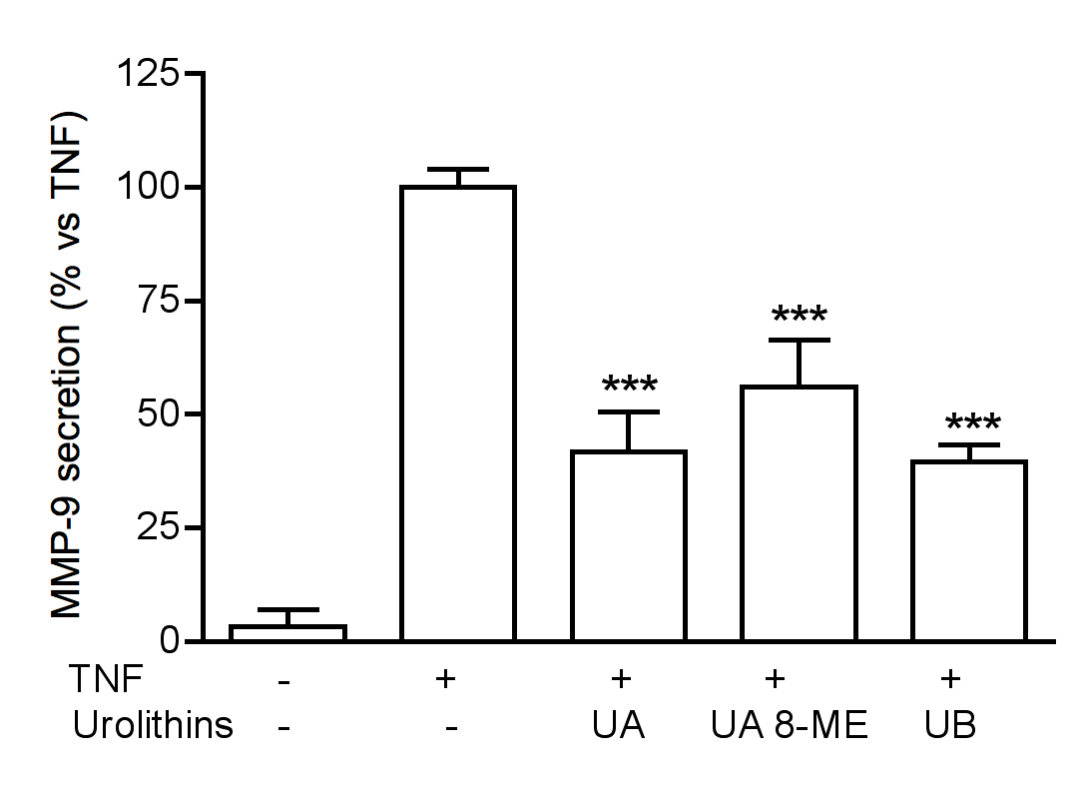
**Effect of urolithins on MMP-9 secretion and mRNA levels in THP-1 cells stimulated with TNF**. Urolithins A, B and 8-ME at 25 μM decreased the secretion of MMP-9 in cells stimulated with TNF (-58, -60 and -44%, respectively). All experiments were reproduced at least three times. Statistical analyses were performed with GraphPad Prism 5 software, using 1-way ANOVA test followed by Bonferroni's post hoc test. The significance was indicated as *** *p *< 0.001.

Urolithins A, B and 8ME at 25 μM inhibited the release of MMP-9 in either Hz or TNF stimulated THP-1 cells, urolithins A and B being the most active (-87, -37, and -74%, respectively; Figure 5A); the effect was associated to a decrease of MMP-9 mRNA levels (-88%, -95% and -82% respectively; Figure 5B). Similarly urolithins A, B and 8-ME decreased the secretion of MMP-9 in cells stimulated with TNF (-58, -60 and -44%, respectively; Figure 6).

### Effect of Pg-FET and individual compounds on the NF-κB system

**Figure 7:**
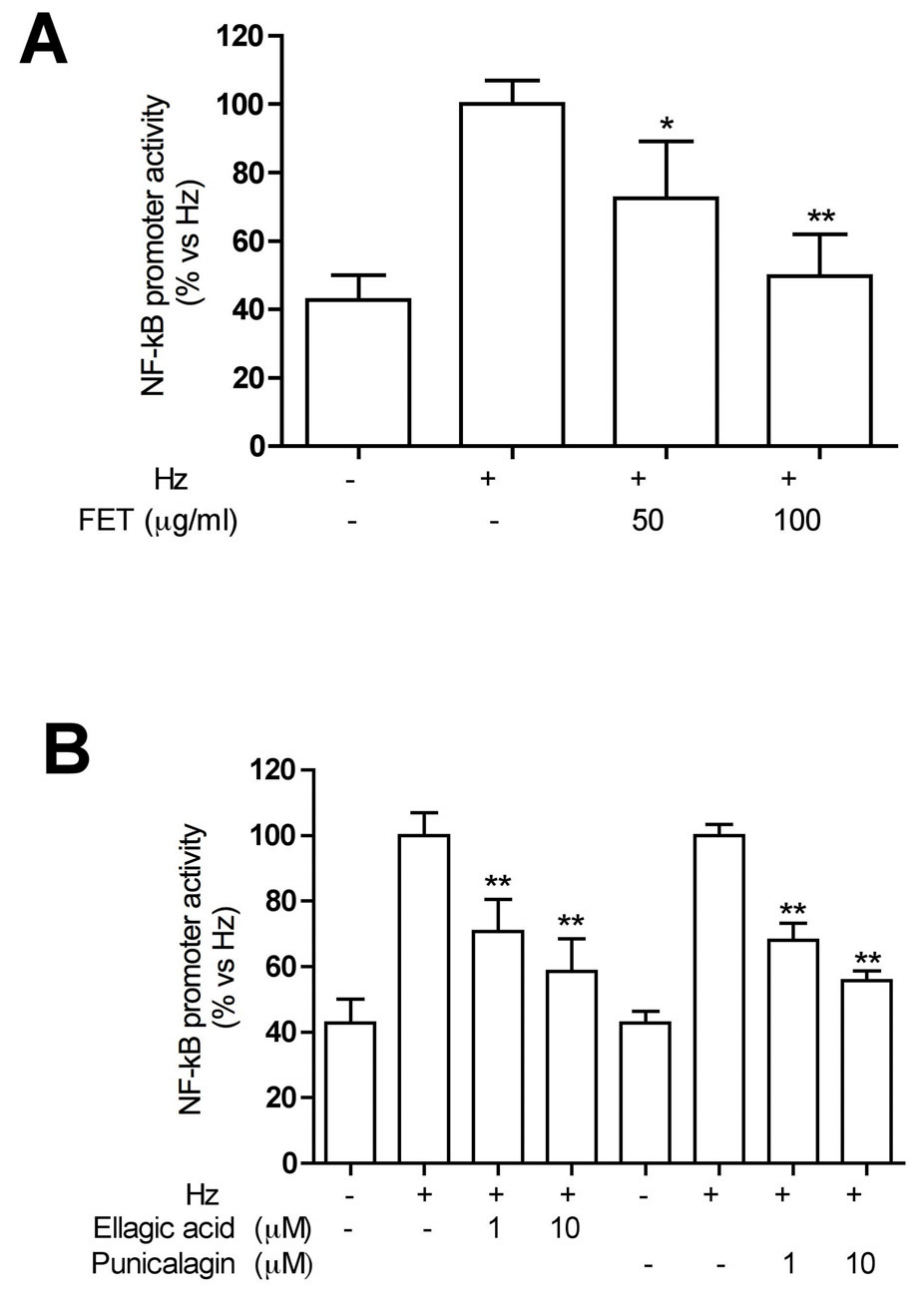
**Effect of *Pg*-FET (panel A) and individual compounds (panel B) on NF-kB promoter activity in THP-1 cells stimulated with Hz**. *Pg*-FET, at 50 and 100 μg/ml, inhibited the Hz-induced NF-κB promoter activity by 28% and 50%, respectively. Both individual compounds at 1-10 μM antagonized the Hz-induced promoter activity: -30% and - 41% for EA; -32% and -45% for punicalagin, respectively. Parthenolide (10 μM) was used as reference inhibitor of the NF-κB (~50% inhibition) promoter activity. Data are expressed as mean ± S.D. of triplicate samples. All experiments were reproduced at least three times and, where indicated, representative experiments are shown. Statistical analyses were performed with GraphPad Prism 5 software, using 1-way ANOVA test followed by Bonferroni's post hoc test. The significance was indicated as follows: * *p *< 0.05; ** *p *< 0.01.

**Figure 8:**
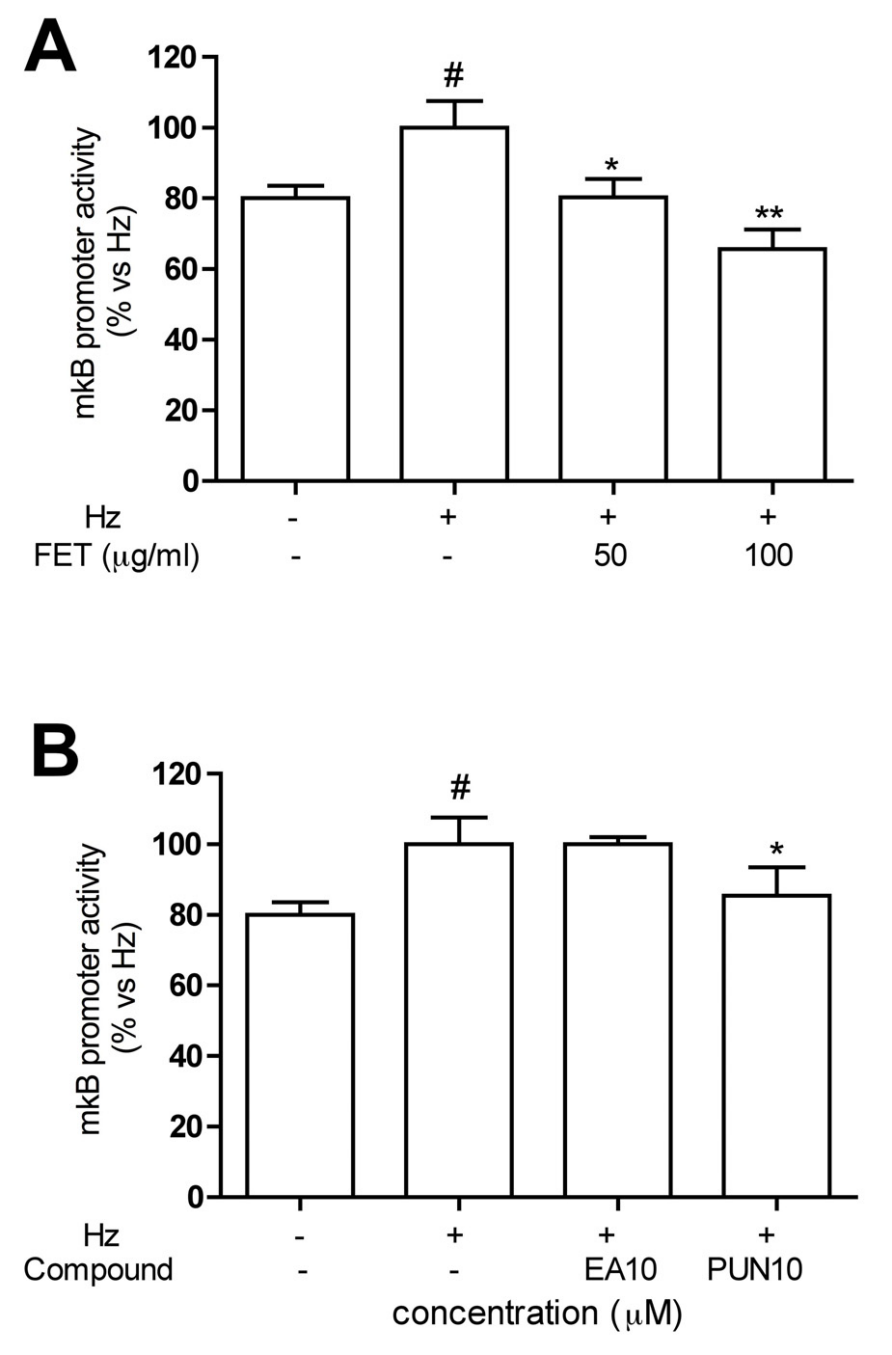
**Effect of *Pg*-FET (panel A) and individual compounds (panel B) on mkB promoter activity (MMP-9 promoter mutated in the kB site) in THP-1 cells stimulated with Hz**. The mutated promoter activity was resistant to Hz induction in comparison to the native promoter (1.3 fold vs 2.5 fold increase) and the inhibitory effect by *Pg*-FET was less pronounced as well (-20% and -35% at 50 and 100 μg/ml, respectively). Punicalagin at 10 μM inhibited the mutated promoter activity by only 25%, and EA was inactive. Parthenolide (10 μM) was used as reference inhibitor of the NF-κB (~50% inhibition) and MMP-9 (~80% inhibition) promoter activity. Data are expressed as mean ± S.D. of triplicate samples. All experiments were reproduced at least three times. Statistical analyses were performed with GraphPad Prism 5 software, using 1-way ANOVA test followed by Bonferroni's post hoc test. The significance was indicated as follows: * *p *< 0.05; ** *p *< 0.01; # *p *< 0.05 vs ctrl without Hz.

To evaluate if *Pg*-FET and individual components inhibited MMP-9 secretion and gene expression by acting on the NF-κB system, THP-1 cells were transiently transfected with a reported plasmid bearing the luciferase gene under the control of a promoter containing NF-κB binding sites. Transfected cell were then stimulated with Hz or TNF. As shown in Figure 7A, *Pg*-FET, at 50 and 100 μg/ml, inhibited the Hz-induced NF-κB promoter activity by 28% and 50%, respectively. Both individual compounds at 1-10 μM antagonized the Hz-induced promoter activity: -30% and - 41% for EA; -32% and -45% for punicalagin, respectively (Figure 7B). To further confirm that the inhibition of MMP-9 promoter activity reflected a down-regulation of the NF-kB system, *Pg*-FET, EA, and punicalagin were tested on THP-1 cells transfected with MMP-9 promoter mutated at the kB binding site (mκB). As reported in Figure 8A, the mutated promoter activity was resistant to Hz induction in comparison to the wild-type (1.3 fold vs 2.5 fold increase) and the inhibitory effect by *Pg*-FET was less pronounced as well (-20% and -35% at 50 and 100 μg/ml, respectively). Consistently with these results, punicalagin at 10 μM inhibited the mutated promoter activity by only 25%, and EA was inactive (Figure 8B).

## Discussion

The sun-dried rind of the immature fruit of *Punica granatum *is used as an herbal anti-malarial remedy. The drug is rich in ellagitannins, namely EA, punicalagin, punicalin and EA glycosides [5]. In a previous work we demonstrated that the fraction enriched in tannins (*Pg*-FET) obtained from the methanolic extract of *Pg *inhibited *in vitro *the growth of *P. falciparum *asexual blood stages. According to previous results and those from other groups, ellagitannins and in particular EA, were identified as the active principles [5,6,28,29]

A phytocomplex, such as a herbal formulation, is characterized by a mixture of chemicals with a wide array of biological effects. The overall clinical effects of a phytocomplex represent a synthesis of several different activities exerted by clusters of molecules acting on different molecular targets. Therefore, it was expected that the anti-malarial activity of *Pg *could be attributed to the action of the components of the phytocomplex on other factors involved in the pathogenesis of the malarial disease and not only on the parasite itself. With this in mind, the possibility that *Pg*-FET could interact with the production of MMP-9, an enzyme directly implicated in the pathogenesis of malaria was considered. MMP-9 expression is higher in mice brain with cerebral malaria [30] and human monocytes fed with Hz or trophozoite-parasitized red blood cells displayed increased activity and protein/mRNA expression of MMP-9 and increased production of TNF [11,12]. The enhancement of MMP-9 activity was causally related to the increase of IL-1 beta production. 15-HETE, one of the monohydroxy derivatives of polyunsaturated fatty acids (OH-PUFAs) attached to Hz, was considered responsible for the increase of IL1-beta production and MMP-9 activity [12].

The present data confirm that Hz or TNF increase the secretion and mRNA levels of MMP-9 in THP-1/monocytes. In addition, it has been shown that *Pg*-FET and individual compounds are able to antagonize such stimulation. The total fraction and the pure compounds inhibited Hz and TNF-induced MMP-9 promoter activity.

Considering that the level of EA and punicalagin measured in *Pg*-FET is 13.4 and 29.1%, the amount of *Pg*-FET necessary to exert this effect is compatible with the concentrations of EA and punicalagin necessary for the inhibition of MMP-9. It can then be concluded that EA and punicalagin greatly contribute to the overall effect exerted by *Pg*-FET.

These two compounds are metabolized *in vivo *by the human intestinal microflora into at least three metabolites named urolithins [16-20]. It has been reported here that urolithins as well diminished MMP-9 secretion and mRNA levels induced by HZ or TNF. The effect could be observed at concentrations of metabolites (25 μM) close to those attained in plasma (18-20 μM) after pomegranate juice consumption [16]. These results suggest that also the metabolites of ellagitannins participate to the control of excessive production of MMP-9 and in turn they may protect against the increased production and function of the noxious cytokine TNF.

To investigate more deeply how *Pg*-ellagitannins affect the signalling cascade leading to MMP-9 expression, the involvement of NF-κB system was investigated. It is known that several cytokines, including TNF, act at different levels ultimately promote the binding of the NF-κB complex to target sequences thus inducing the transcription of several genes, among which the MMP-9 gene. As in many other situations, in Hz-fed murine macrophages, TNF up-regulation is linked to the activation of the NF-κB pathway [31,32]. As already shown by Prato et al in human monocytes [12], Hz increased TNF levels in THP-1 cells [33]. By showing that *Pg*-FET and its ellagitannins inhibited NF-κB promoter activity and that the effect was evident only when the wild type promoter was used and less pronounced when the promoter was mutated for the NF-κB binding site, it has been demonstrated that *Pg*-FET and ellagitannins block NF-κB-driven transcription thus affecting the entire cytokine cascade. However, it could be predicted that their anti-inflammatory role goes beyond the effects on MMP-9.

In addition, ellagitannins and their hydrolysis products, i.e. EA, have been shown to inhibit the activation of inflammatory pathways including but not limited to the NF-κB system [34-36]. Thus, additional mechanisms of action could also be considered, such as mitogen-activated protein kinases (MAPK), which are modulated by pomegranate [35,37,38]. The peroxidation of the lipid components (HETEs) attached to Hz has been suggested to be instrumental for enhanced MMP-9 activity [12]. *Pg*-FET is a mixture of polyphenols with high antioxidant capacity that are likely capable to prevent the non-enzymatic oxidation of PUFAs to hydroperoxides.

## Conclusions

In conclusion, the beneficial clinical effects of the fruit rind of *Punica granatum *for the treatment of malaria possibly include the direct anti-parasitic activity and the ability to limit the excess inflammatory response of the host, thus limiting the risk of progression to the more severe form of the disease, including the onset of cerebral malaria.

## List of abbreviations

(FET): Fraction enriched in tannins; (MMP-9): metalloprotease-9; (*Pg*): *Punica granatum *L.; (OMARIA): Orissa Malaria Research Indigenous Attempt; (IRBC): infected red blood cells; (*Pf*): *Plasmodium falciparum *; (EA): Ellagic acid; (PMA): Phorbol myristate acetate; (Hz): Haemozoin; (FCS): foetal calf serum.

## Competing interests

The authors declare that they have no competing interests.

## Authors' contributions

MDA and GVG designed the research. GVG, MDA and MB performed the experiments. NB helped with the preparation of Hz. DB supplied the plant and contributed to discussion of the data. MDA, DT and EB drafted and wrote the final manuscript. SR synthesized urolithins. All authors approved the final manuscript.
